# Spin‐Decoupled Transflective Spatial Light Modulations Enabled by a Piecewise‐Twisted Anisotropic Monolayer

**DOI:** 10.1002/advs.202202424

**Published:** 2022-06-04

**Authors:** Rui Yuan, Chun‐Ting Xu, Han Cao, Yi‐Heng Zhang, Guang‐Yao Wang, Peng Chen, Yan‐Qing Lu, Wei Hu

**Affiliations:** ^1^ National Laboratory of Solid State Microstructures Key Laboratory of Intelligent Optical Sensing and Manipulation and College of Engineering and Applied Sciences Nanjing University Nanjing 210023 China

**Keywords:** liquid crystal, planar optics, spatial light modulation

## Abstract

Wavefront control lies at the heart of modern optics. Metasurfaces with specifically tailored resonators can encode different phases to two orthogonal polarization components, but suffer from wavelength‐dependent efficiency, sophisticated fabrication, and limited size. Liquid crystals, another excellent candidate for planar optics, are restricted to spin‐coupled conjugated phase modulations. Planar optics with spin‐decoupled functions is expected to release the multifunctionality of modern optics. Here, a spin‐decoupled transflective spatial light modulator is presented with a piecewise‐twisted anisotropic monolayer. The phases of reflected and transmitted light can be independently customized by preprogramming the initial orientations of the periodic helix and mirror‐symmetric dual‐twist configuration, respectively. A transflective orbital angular momentum encoder and decoder is demonstrated, which is simultaneously compatible with different multiplexing techniques. This work releases the multifunctionality of advanced planar optics and may upgrade existing devices in optical informatics.

## Graphical abstract

A spin‐decoupled transflective spatial light modulator is presented with a piecewise‐twisted anisotropic monolayer. The phases of reflected and transmitted light can be independently customized by preprogramming the initial orientations of the periodic helix and mirror‐symmetric dual‐twist configuration, respectively. This work releases the multifunctionality of advanced planar optics and may upgrade existing devices in optical informatics.

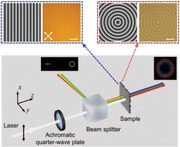

## 1 Introduction

Wavefront control plays a vital role in optics, which is developing toward miniaturization, integration, and multiple functions. A metasurface is composed of plasmonic or dielectric artificial nanostructures in an ultrathin plane. It overcomes the bulky size of traditional deflective and diffractive optics based on propagation phase accumulation, and significantly promotes the development of modern optics.^[^ ^1^, ^2^, ^3^, ^4^ ^]^ Recently, different phase diagrams have been separately encoded to two orthogonal polarization components based on spatially tailoring the geometries of resonators. By this means, multifunctionalities such as distinguishing hologram generations,^[^ ^5^ ^]^ multimode imaging,^[^ ^6^ ^]^ tunable lenses,^[^ ^7^ ^]^ and different specific optical field conversions^[^ ^8^ ^]^ have been demonstrated. However, the wavelength‐dependent efficiency, sophisticated fabrication, and limited size of common metadevices drastically hinder their practical usage. New approaches for spin‐decoupled spatial light modulation with broadband high efficiency and cost‐efficient volume production are highly sought after in broad fields, including optical computing, optical communications, virtual/augmented reality, and holographic displays.

Liquid crystals (LCs) are considered as another excellent candidate for planar optics due to their intrinsic broadband birefringence and various external‐field responsiveness.^[^ ^9^ ^]^ LC on silicon^[^ ^10^ ^]^ and photopatterned geometric phase optical elements^[^ ^11^ ^]^ enable the accurate and free control of LC directors, thus providing powerful approaches for spatial light manipulation. In these cases, the maximum efficiency only occurs at the half‐wave condition, and it drops significantly when the wavelength deviates. Helical configurations, which are easily generated in self‐organized systems but illusive in artificial microstructures, have been introduced to solve this problem. Cholesteric LC (CLC) features a chiral helical structure.^[^ ^12^ ^]^ In a photopatterned planarly aligned cell, CLC self‐assembles into ordered chiral superstructures and exhibits unique features. The light within the Bragg refection band and with the same helicity of CLC is reflected in equal high efficiency, and encoded with certain geometric phase owing to the photonic spin–orbit interaction.^[^ ^13^, ^14^, ^15^ ^]^ Moreover, the Bragg–Berry phase change is twice the initial orientation of the CLC helixes. Thus, a spin‐determined polychromatic phase modulation is exhibited. Meanwhile, the light with opposite helicity transmits and just experiences a uniform dynamic phase change. In addition to broadband reflective geometric phase manipulation, the twist configuration is also adopted to extend the bandwidth of transmissive geometric phase devices. The self‐aligned multitwist structure of chiral reactive mesogen can compensate for the dispersion of birefringence and thus meets half‐wave conditions in a much broader bandwidth than traditional nontwisted nematics.^[^ ^16^, ^17^, ^18^ ^]^ However, to date, all LC‐based geometric phase optics has been restricted to spin‐coupled conjugated phases. Planar optics with spin‐decoupled functions is thus an urgent pursuit and expected to release the multifunctionality of modern optics.

Here, a solution for spin‐decoupled transflective spatial light modulation is proposed and demonstrated via piecewise tailoring the helicity of an anisotropic monolayer in the light propagation direction and customizing the space‐variant initial orientations of helixes in the cross‐section of light propagation. The monolayer is composed of a periodic helix and a mirror‐symmetric dual‐twist configuration. The first part splits the orthogonal spins to opposite directions and encodes one geometric phase to the reflected light, while the second part encodes the other geometric phase to the transmitted light. Arbitrary phases can be endowed by point‐to‐point photoaligning the two different parts separately. By this means, a polarization grating and an axicon lens are simultaneously encoded. As a result, (93.97 ± 1.44)% right‐handed circular polarization in the range of 560–600 nm is deflected, while (97.29 ± 1.06)% of opposite polarization in the range of 450–800 nm is diffracted to concentric circles accompanied by a handedness conversion. Transflective orbital angular momentum (OAM) encoder and decoder is further demonstrated. It releases the multifunctionality of planar optics with the merits of broadband high efficiency as well as easy and cost‐efficient fabrication, and may upgrade many fantastic applications in optical computing, communicating, imaging, and information displays.

## 2 Experiments and Results

### 2.1 Principle and Characterization of the Piecewise‐twisted Anisotropic Monolayer

**Figure 1:**
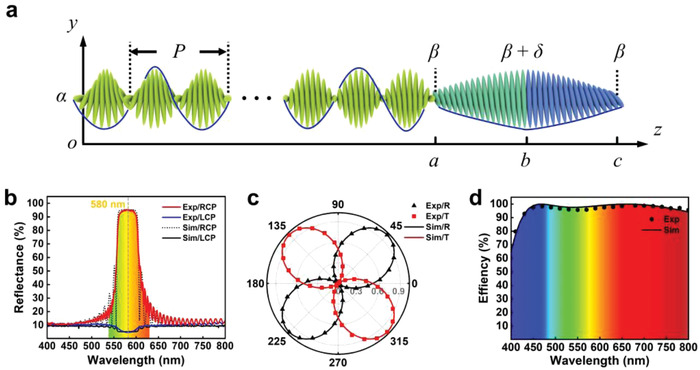
Illustration and characterization of the piecewise‐twisted anisotropic monolayer. a) Schematic illustration of the piecewise‐twisted anisotropic monolayer with *α* = 0° and *β* = 15°. b) Wavelength and circular polarization dependent reflections of the monolayer. c) Dependencies of transmission and reflection on incident polarization at 580 nm. d) Wavelength dependent circular polarization conversion efficiency of the monolayer.

As illustrated in **Figure** **1**a, all LC directors of the piecewise‐twisted anisotropic monolayer are parallel to the surfaces (*x*–*y* plane), while the variation in azimuthal angle *φ* along the *z‐*axis is depicted by a piecewise function as

(1) $$\phi =\left\{\begin{array}{cc} \frac{2\pi }{P}z+\alpha , & 0\leq z<a\\ \beta +\frac{\delta }{b-a}\left(z-a\right), & a\leq z<b\\ \beta +\delta -\frac{\delta }{c-b}\left(z-b\right), & b\leq z\leq c \end{array}\right.$$

A periodic helix exists in the range of 0–*a*. *P* and *α* are the pitch and initial orientation to the *x*‐axis of the helix. *a* is an integral multiple of *P*/2 and is set over 10*P* to maximize the Bragg reflection. For circular polarization of the same helicity to the structure, the wavelength *λ* falling in the Bragg regime *n*_o_ *P* < *λ* <*n*_e_ *P*, where *n*_o_ and *n*_e_ are the ordinary and extraordinary refractive indices of the LC, is selectively reflected. Ranges of *a*–*b* and *b*–*c* jointly form a mirror‐symmetric dual‐twist configuration. *β* is the orientation of LC to the *x*‐axis in the *x*–*a*–*y* and *x*–*c*–*y* planes. *δ* is the twist angle between the LC directors of the *x*–*b*–*y* and *x*–*a*–*y* planes.

For a beam with a vector along *z*‐axis, corresponding reflection can be calculated according to Berreman's 4 × 4 matrix.^[^ ^19^ ^]^ Only LCs in 0–*a* are considered, which make a full contribution to the spin selective reflection. We set *α* = 0°, *P* = 410 nm, *a* = 17*P*, *n*_o_ = 1.55, *n*_e_ = 1.67, and the simulated results are shown in Figure 1b. For right circular polarization (RCP, photon spin of −ℏ) of the same helicity to the structure, a Bragg reflective band with a center wavelength of 580 nm is obtained (black dashed line). No peak is observed for left circular polarization (LCP, photon spin of +ℏ) (black solid line). We measure the circular polarization dependent reflection of a uniformly aligned piecewise‐twisted anisotropic film, and the experimental results (red and blue solid lines for RCP and LCP, respectively) are in good agreement with the simulations. (93.97 ± 1.44)% of RCP light is reflected in the range of 560–600 nm, while the reflection of LCP light is suppressed to (5.54 ± 0.74)%. We record the reflection and transmittance of a 580 nm beam during continuously varying the angle between an achromatic quarter‐wave plate (AQWP) and the incident linear polarization. As shown in Figure 1c, the light is totally reflected by the film at 45° and 225° (RCP), while the light totally transmits at 135° and 315° (LCP) in both simulations and experiments. Via altering the incident polarization, the energy distribution between reflection and transmission can be gradually tuned, vividly showing the spin‐dependent characteristics of the piecewise‐twisted anisotropic film.

The mirror‐symmetric dual‐twist configuration is introduced to realize broadband *π* retardation compensation. Its polarization conversion efficiency *η* is calculated by^[^ ^16^ ^]^

(2) $$\begin{array}{c} \eta =1-{\left(\cos^{2}\sqrt{\delta^{2}+{\left(\pi \Delta nd/\lambda \right)}^{2}}+\left[\delta^{2}-{\left(\pi \Delta nd/\lambda \right)}^{2}\right]\frac{\sin^{2}\sqrt{\delta^{2}+{\left(\pi \Delta nd/\lambda \right)}^{2}}}{\delta^{2}+{\left(\pi \Delta nd/\lambda \right)}^{2}}\right)}^{2} \end{array}$$

where Δ*n* is the birefringence of the LC and *d* is the thickness of *ab* and *bc*. We set *δ* = 65°, *d* = 1542 nm and Δ*n* = 0.134 + 8832/*λ* ^2^ + 1.337 × 10^8^/*λ* ^4^, which is a Cauchy's dispersion formula fitting to the birefringence of the adopted LC. The simulated *η* is shown by the black solid line in Figure 1d. The mirror‐symmetric dual‐twist configuration exhibits a high *η* in the whole visible band. When broadband LCP light incidents, the other pair of AQWP and polarizer of −45° is used to measure *η*, which is defined as the ratio of the detected intensity to the total transmittance. The experimental results (hexagonal point) reveal (97.29 ± 1.06)% in the range of 450–800 nm, which is in good agreement with the simulations.

### 2.2 Transflective Spatial Light Modulations

**Figure 2:**
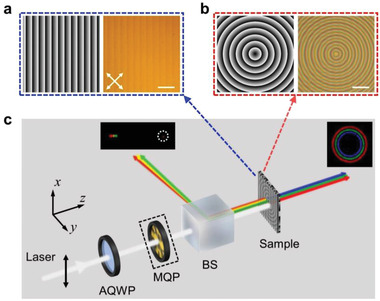
Transflective spatial light modulator. Phase diagrams (left, black to white indicates 0 to 2*π*) and images (right, all scale bars indicate 100 µm, and the white arrows reveal the directions of crossed polarizers) of a) polarization grating and b) axicon lens. c) The optical setup for the measurements. AQWP, achromatic quarter‐wave plate; MQP, meta‐*q*‐plate; BS, beam splitter.

Since the encoded geometric phase is twice the initial orientation of the piecewise helixes and the spin is split by the periodic helix, spin‐decoupled spatial light modulations can be carried out via spatially programming *α* and *β*, respectively. For easy demonstration, the phase diagrams of a polarization grating and an axicon lens are separately recorded to *α* and *β*. *α* = *πx*/*Λ*_1_ and *β* = *πr*/*Λ*_2_, where *Λ*_1_ and *Λ*_2_ are the periods of the polarization grating and the axicon lens, respectively, and$r=\sqrt{x^{2}+y^{2}}$. **Figure** **2**a,b shows the phase diagrams of a polarization grating and an axicon lens with *Λ*_1_ = *Λ*_2_ = 39 µm. Figure 2a also reveals an image of the anisotropic film in reflection mode under a polarization optical microscope (POM) with a pair of crossed polarizers. A periodic brightness change consistent with the phase diagram is observed due to the original‐helix‐orientation *α* dependent Fresnel reflection at the surface of the helical Bragg layer. Figure 2b shows a transmission‐mode image of the inverted sample. Here, the mirror‐twist layer works as a broadband halfwave plate, and it exhibits a fourfold rotation as traditional waveplate. Thereby, under a POM, twice denser concentric rings compared to the phase design are observed due to the *π* variation of optical axis *β* in a single pitch. Noticeably, the polarization grating is indistinctly observed as well at the transmission mode.

The transflective spatial light modulation performance of the sample is characterized by the optical setup illustrated in Figure 2a. A linearly polarized supercontinuum laser transmits an AQWP to generate RCP light. The reflected light is encoded with the geometric phase of the designed polarization grating, and RCP light within the Bragg band (red, yellow, and green for instances) is deflected to different angles consistent with the grating equation. As shown in the inset of Figure 2c, the 0th order (marked by the white dashed circle) is very weak, verifying the high efficiency of the phase modulation. When the orthogonal spin is incident, the geometric phase of the designed axicon lens is encoded. It can also be considered as circular polarization grating, and thus the transmitted light is diffracted to different concentric rings. Red, green, and blue light exhibit equal‐high efficiency due to the transmissive broadband characteristic of the sample. Therefore, spin‐decoupled spatial phase modulation is demonstrated. When linearly polarized light within the working band incidents, the orthogonal spins are split into opposite directions and encoded with separate phases according to the predesigned distributions of *α* and *β*, respectively.

### 2.3 Transflective OAM Encoding

Photons of an optical vortex (OV) with a helical phase‐front carry OAM of *m*ℏ, where *m* is the topologic charge.^[^ ^20^ ^]^ Thanks to the theoretically infinite number of *m*, OAM can be adopted as extra informatic channels, i.e., OAM‐based mode‐division multiplexing (MDM), in addition to the existing wavelength‐division multiplexing (WDM) and polarization‐division multiplexing (PDM) technologies.^[^ ^21^, ^22^ ^]^ The combination will drastically enhance the capacity of optical communications.^[^ ^23^ ^]^ Devices compatible simultaneously with MDM, WDM, and PDM are thus highly pursued. The unique function of the polychromatic and spin‐decoupled phase modulations of the proposed piecewise‐twisted anisotropic monolayer perfectly suits the above requirement. Therefore, the technique is reasonably expected to significantly upgrade existing communication systems. Herein, we specifically design a multiplexing phase hologram (MPH)^[^ ^24^ ^]^ and a Dammann vortex grating (DVG)^[^ ^25^ ^]^ to demonstrate a spin‐decoupled transflective OAM encoder.

**Figure 3:**
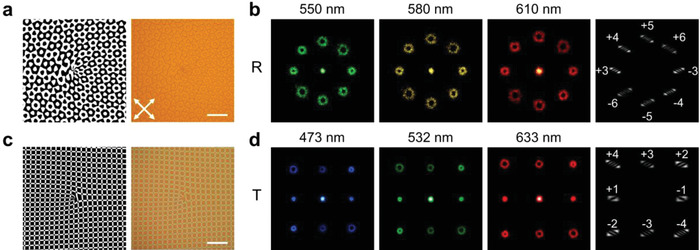
Transflective OAM encoder. The phase diagrams (left, black indicate 0 and white indicate *π*) and corresponding images (right) of a) an MPH and c) a DVG. All scale bars indicate 100 µm. The white arrows reveal the directions of crossed polarizers. b) Reflected diffraction patterns of 550, 580, and 610 nm light and corresponding OAM detections (right). d) Transmitted diffraction patterns of 473, 532, and 633 nm light and corresponding OAM detections (right).

Here, the MPH is a pure phase mask that is a superposition of multiple 1D vortex gratings along different directions. The phase of such an MPH can be expressed as

(3) $$\varphi_{\mathrm{MPH}}\left(x,y\right)=\mathrm{Binary}\left[\arg\left(\sum_{j}\omega_{j}\exp\left\{i\left[m_{j}\theta +\left(x\cos\sigma_{j}+y\sin\sigma_{j}\right)\frac{2\pi }{\Lambda_{j}}\right]\right\}\right)\right]$$

where *j* is the number of 1D vortex gratings, *σ_j_* is the direction of the 1D vortex gratings with respect to *x*‐axis, *ω*_*j*_ is the power weight coefficient of each OV, *m_j_* is the topological charge, *Λ_j_* is the period of grating and *θ* = actan(*y*/*x*). The function of Binary[] is via setting the positions and number of 0‐*π* phase transition points,^[^ ^26^ ^]^ to obtained a series of equal‐energy OVs. We set *α* = *ϕ*_MPH_/2, *σ_j_* = {0°, 45°, 90°, 135°}, *m_j_* = {3, 4, 5, 6} and normalized 0‐*π* phase transition point *x* = 0.5; thus, a series of equal‐energy OVs carrying topological charges of {−6, −5, −4, −3, +3, +4, +5, +6} will be obtained at objective orders. **Figure** **3**a shows the phase diagram of the MPH, and the corresponding image is consistent with the design. Figure 3b reveals the reflected octagonal OV arrays at 550, 580, and 610 nm. The radius of OV is proportional to |*m*| and different colors are diffracted to different angles. *m_j_* is identified by an anastigmatic transformation method.^[^ ^27^ ^]^ The numbers and tilt directions of the generated dark stripes indicate the corresponding topological charges, which are labeled in Figure 3b.

2D DVG combines a horizontal and a vertical 1D DVGs. Its transmission function is expressed as

(4) $$\begin{array}{cc} T\left(x,y\right) & =\exp\left(i\varphi_{\mathrm{DVG}}\left(x,y\right)\right)\\ & =\sum_{k_{1}}\sum_{k_{2}}\omega_{k_{1}}\omega_{k_{2}}\exp\left[\begin{array}{c} i\left(k_{1}m_{1}+k_{2}m_{2}\right)\theta \\ +i2\pi \left(\frac{k_{1}}{\Lambda_{x}}x+\frac{k_{2}}{\Lambda_{y}}y\right) \end{array}\right] \end{array}$$

where *ϕ*_DVG_ is the phase function of the 2D DVG, $\omega_{k_{1}}$ and $\omega_{k_{2}}$ are the power weight coefficients of each OV, *k*_1_ and *k*_2_ are the diffraction orders of horizontal and vertical 1D DVGs, *m*_1_ and *m*_2_ denote corresponding topological charges, *Λ_x_* and *Λ_y_* are periods of grating in the *x* and *y* directions, respectively.^[^ ^28^ ^]^ Here, we set *β* = *ϕ*_DVG_/2 and the normalized 0‐*π* phase transition point *x* = *y* = 0.73526, a 3 × 3 DVG with *m*_1_ = −1 and *m*_2_ = 3 is recorded in *β*. Figure 3c depicts the phase diagram of the 3 × 3 DVG, and the corresponding image is consistent with the design. Figure 3d reveals the transmitted 3 × 3 tetragonal OV arrays at 473, 532, and 633 nm. Corresponding topological charges are identified and labeled in Figure 3d as well.

### 2.4 Transflective OAM Decoding

**Figure 4:**
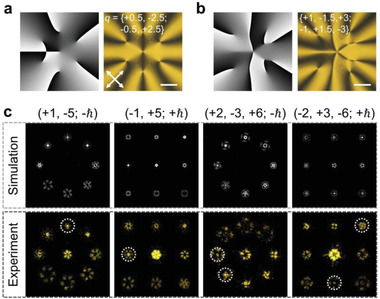
Transflective OAM decoder. The phase diagrams (left, grayscale image, black to white indicates 0 to 2*π*) and corresponding images of meta‐*q*‐plate with a) *q* = {+0.5, −2.5; −0.5, +2.5} and b) *q* = {+1, −1.5, +3; −1, +1.5, −3}. All scale bars indicate 100 µm. The white arrows reveal the directions of crossed polarizers. c) Simulated and measured OAMs decoding at 580 nm. All recovered modes are marked in dashed circles.

An OV will recover to a Gaussian beam when it is diffracted to the order with opposite topological charge. It enables a way for OAM detection, i.e., OAM decoding. When mixed OAM states are diffracted to the designed orders, OVs with opposite *m* to the objective orders recover to Gaussian beams.^[^ ^28^ ^]^ This phenomenon can be utilized for simultaneously decoding OAMs. For the above OAM coder, the set for spin and OAM detection is depicted as {±3, ±4, ±5, ±6; −ℏ} and {±1, ±2, ±3, ±4; +ℏ}. Here, two OVs of mixed OAM states are generated by meta‐*q*‐plates.^[^ ^11^, ^29^ ^]^ Their phase diagrams are exhibited in **Figure** **4**a,b, with corresponding images shown on the right. OVs with *m* = {+1, −5} for −ℏ & {−1, +5} for +ℏ and *m* = {+2, −3, +6} for −ℏ & {−2, +3, −6} for +ℏ are generated by the two meta‐*q*‐plates, respectively. The meta‐*q*‐plate is put in the setup shown in Figure 2a. A 580 nm laser transmits it and thus carries designed mixed OAMs with spin conversion simultaneously. The far‐field diffraction patterns are calculated via angular spectrum theory^[^ ^30^ ^]^ and the corresponding results are shown as the upper row in Figure 4c. The measured results are consistent with the simulations. States of {+1, −5; −ℏ} & {−1, +5; +ℏ} and {+2, −3, +6; −ℏ} & {−2, +3, −6; +ℏ} are detected and shown as the bottom row in Figure 4c. Noticeably, cases not included in the detection set cannot be decoded, such as the {+1; −ℏ}, {+5; +ℏ}, {+2; −ℏ}, and {−6; +ℏ} states in the measurement.

## 3 Discussion

The monolayer is composed of a periodic helix and a mirror‐symmetric dual‐twist configuration. The working band can be rationally tailored by adjusting the twist of the configuration. The reflective Bragg band can be shifted by tuning the pitch of the periodic helix via adjusting the concentration or helical twist power of the chiral dopant. Here, the Bragg reflection band is narrower than the transmissive working band. This issue can be well addressed by introducing larger birefringent LCs^[^ ^31^ ^]^ and gradient‐variant helix,^[^ ^32^ ^]^ as well as stacking a multipitch helical layers.^[^ ^33^ ^]^ Moreover, by optimizing the half‐wave condition of the mirror‐symmetric dual‐twist configuration, the transmissive band can be shifted to the telecom band. Superbroadband can be realized by further adopting a four‐twist configuration.^[^ ^18^ ^]^ Due to the high resolution and excellent flexibility of the photoalignment technique, arbitrary phase diagrams can be recorded to *α* and *β* in a point‐to‐point manner. For easy demonstration, a small topological charge scale transflective OAM encoder and decoder is presented. Further extending the scale and shifting the working wavelength to telecom band is feasible. It thus supplies a practical platform for optics simultaneously compatible with MDM, WDM, and PDM.

The unique properties of broadband high efficiency and spin‐decoupled transflective spatial light modulation originate from the self‐organization nature of LCs, making the technique easy and cost‐efficient. The relationship between the configuration of the anisotropic monolayer and the performance of generated elements is clear and a general framework for transflective modulator designing is presented, leading to everything predictable. Compared to the conventional multifunction achieved with multiple elements, the monolithic film strategy is compact, more material and power efficient. Additionally, the precisely adjusting among separate elements and interferences among light reflected by various surfaces are totally avoided. The minimum controllable unit here is ≈1 µm^2^ in size and the resolution can be further improved via adopting higher resolution projection, interference or direct laser writing systems. The theoretical resolution is up to the diffraction limit. It is comparable with the feature size of dielectric metasurfaces, meeting the high‐standard requirement of the optical phase modulations. The obtained performances will perfectly match or even exceed those generated by traditional ways. Therefore, this work may significantly upgrade the multifunctionalities of planar optics, which may find wide applications in computing, communicating, imaging, and information displays.

## 4 Conclusion

Spin‐decoupled transflective spatial light modulations are demonstrated with a piecewise‐twisted anisotropic monolayer. The film consists of a periodic helix and a mirror‐symmetric dual‐twist configuration. Via tailoring the helicity of the monolayer along the light propagation direction, both the reflection and transmissive bands can be freely manipulated. Through point‐to‐point photoaligning the space‐variant initial orientations of the helixes (*α* and *β*), arbitrary phases can be endowed to the oppositely propagated light selected by spins. Simultaneous spin and OAM encoding and decoding are demonstrated in a broadband. It supplies a practical method for MDM, WDM, and PDM compatible optics, meeting the urgent demand in optical informatics. In addition to broadband high‐efficiency and spin‐decoupled spatial light modulation, it also exhibits merits of easy and cost‐efficient fabrication, and suitable for volume production. Thus, it may upgrade planar optics and inspires many fantastic applications.

## 5 Experimental Section

### Materials

The photoalignment agent SD1 (DIC Co., Japan) was dissolved in *N*,*N*‐dimethylformamide (0.5 wt%). The LC for the periodic helix contains monomer RM257 (HCCH Co., China), chiral dopant R5011 (HCCH Co., China) and photo‐initiator Irgacure 651 (BASF Co., Germany) with a weight ratio of 97.6:2.1:0.3. LC mixtures for the mirror‐symmetric dual‐twist configuration were prepared by 99.1 wt% LC polymer OCM‐A1 (Raito Materials Technology Co., China) and 0.9 wt% chiral dopant R811/S811 (HCCH Co., China), respectively. The two LC mixtures were dissolved in propylene glycol methyl ether acetate at a concentration of 30 wt%. OCM‐A1 (30 wt%) without chiral dopant was used for meta‐*q*‐plate fabrication.

### Sample Fabrication

The SD1 solution was spin‐coated onto clean glass substrates at 800 rpm for 5 s and then 3000 rpm for 30 s, and annealed at 100 °C for 10 min to form alignment films. Two substrates were separated by 7 µm spacers and sealed to form a cell. A dynamic photopatterning technique with multistep partly overlapping exposure was adapted to record the desired alignments.^[^ ^34^ ^]^ Subsequently, the cell was filled with an RM257/R5011/Irgacure 651 mixture at 90 °C and then cured under UV for 30 min at 0.6 mW cm^−2^. After polymerization, one substrate was separated. The SD1 solution was spin‐coated onto the surface of the film again to form a new alignment film. The other photopatterning was carried out to record the second alignment. Then, LC mixtures of OCM‐A1/R811 and OCM‐A1/S811 were spin‐coated at 1450 rpm once for 80 s and at 2100 rpm twice for the same time, respectively. After each spin‐coating, the sample was heated to 68 °C and cured under UV irradiation at 20 mW cm^−2^ for 2 min. Finally, the piecewise‐twisted anisotropic monolayer was obtained. OCM‐A1 without chiral dopant was spin‐coated twice at 2150 rpm for 40 s on pre‐photopatterned glass substrates to produce meta‐*q*‐plates optimized for 580 nm. After each spin‐coating, the sample was heated to 68 °C and cured under UV irradiation at 20 mW cm^−2^ for 2 min.

### Characterization

The reflection was measured with a spectrometer (NOVA, Ideaoptics Co., China) paired with a light source (iDH2000, Ideaoptics Co., China). RCP and LCP were generated by setting the optical axis of AQWP ±45° with respect to the direction of the polarizer. POM images were captured on a POM (Nikon 50i, Japan) with crossed polarizers. All images were recorded by a CCD camera (Nikon DS‐Ri1, Japan). Diffraction patterns were captured by a digital camera (EOSM, Canon, Japan).

### Statistical Analysis

For reflective spectrometers, the dark state was mearured with no incident light and set as 0%, the total incident energy of polarized light reflected by a mirror (BB1‐E02, Thorlabs, USA) was corresponding to the bright state and set as 100%. For transmitance measurements, light shut state was defined as 0% and total incidence was defined as 100%. The transmission and reflection were calculated by the value of detected intensity reducing the intensity of dark state and divided by the intensity difference of the bright and dark states. In Figure 1c the transmittance and reflectance were normalized according to corresponding maximum and minimum values. The polarization conversion efficiency was defined as the ratio of the detected intensity to the total transmittance. All spectrometers were redrawed by Origin. Matlab was used to perform the simulations and generate the phase diagrams.

## Conflict of Interest

The authors declare no conflict of interest.

## Data Availability

The data that support the findings of this study are available from the corresponding author upon reasonable request.
